# Dual-functional tunable coding metasurface based on saline water substrate

**DOI:** 10.1038/s41598-018-20532-9

**Published:** 2018-02-01

**Authors:** Lei Chen, He Liang Ma, Xiao Jun Song, Ying Ruan, Hao Yang Cui

**Affiliations:** 1 College of Electronics and Information Engineering, Shanghai University of Electric Power, Shanghai, 200090 China; 2 Giantec Semiconductor Incorporated, Shanghai, 201203 China

## Abstract

In this paper, a dual-functional tunable coding metasurface is presented at X band based on water substrate, which can realize two different functions of specific scattering pattern and absorption at two different frequency ranges. Besides, by changing the salinity of the saline water substrate, the absorption performance in high frequency can be tuned, while the scattering pattern in low frequency remains unchanged. A coding element is designed with small water cavity in it. Three coding sequences with different radiation patterns are designed to verify these functions, and one of them is fabricated and measured. Experimental results have good accordance with our simulations, which demonstrates our schemes. We believe this work can not only broaden our design manner of metasurfaces, but also have plenty potential applications in biological and medical detection domain.

## Introduction

Evolved from metamaterials, metasurfaces are two-dimensional (2D) subwavelength element arrays, with superior performance on tailoring the electromagnetic (EM) waves^1,2^. Comparing to the three-dimensional (3D) structure of metmaterials, metasurfaces not only have significantly low profile^3^, but also can be manufactured easily. Besides, by applying flexible substrate, metasurfaces can realize conformal feature^4^, which is more suitable for realistic applications. Due to its superior specialties, plenty of applications using metasurfaces have been proposed, such as holograms^5,6^, cloaks^3,4^, directional couplers for surface plasmon polaritons^7^, orbital angular momentum beams^8^ and so on.

Recently, the concept of coding metasurfaces is proposed^9^, which develops a new manner to design metasurfaces. Different from the conventional metasurfaces, coding metasurfaces focus on discretizing the element sizes. The element phase responses of coding metasurfaces like “0°” and “180°” can be regarded as digitally “0” and “1”^10^, which simplifies the design manner of metasurfaces remarkably. Based on this method, numerous applications have been presented^9–16^, such as multi-bit coding metasurfaces to control EM waves^9^, RCS reduction^9,11^, holograms^16^.

As one of the most abundant and nature-friendly material on Earth, water has strong frequency dispersion at microwave frequencies^17^, which provides plentiful opportunities in microwave designs^18^. Some metamaterials based on water substrate have been presented^19,20^. And some tunable metasurfaces based on water substrate have also been proposed, such as mechanically and thermally tunable absorber^21,22^. However, the works mentioned above neglect another important specialty of water substrate, the salinity of aqueous solution substrate. Since the salinity of solution mainly determines the conductivity, it can affect the permittivity obviously^22^.

In this work, we propose a tunable coding metasurface with two functionalities, which can be selected in frequency domain. The specific coding pattern can be designed to emit random scattering beams at 7 GHz, while at 10 GHz, the metasurface reveals the tunable absorbing performance, mainly determined by the salinity of the saline water substrate. The salinity of solution substrate provides a new degree of freedom to design tunable coding metasurfaces. Our novel design not only integrates two functionalities, but also combines two manipulating methods (frequency and salinity). We believe this work will have plenty potential applications in radar and detecting fields. Besides, based on the EM peculiarity of water substrate, coding metasurfaces may develop various potential applications in biological and medical detection region.

## Results

**Figure 1:**
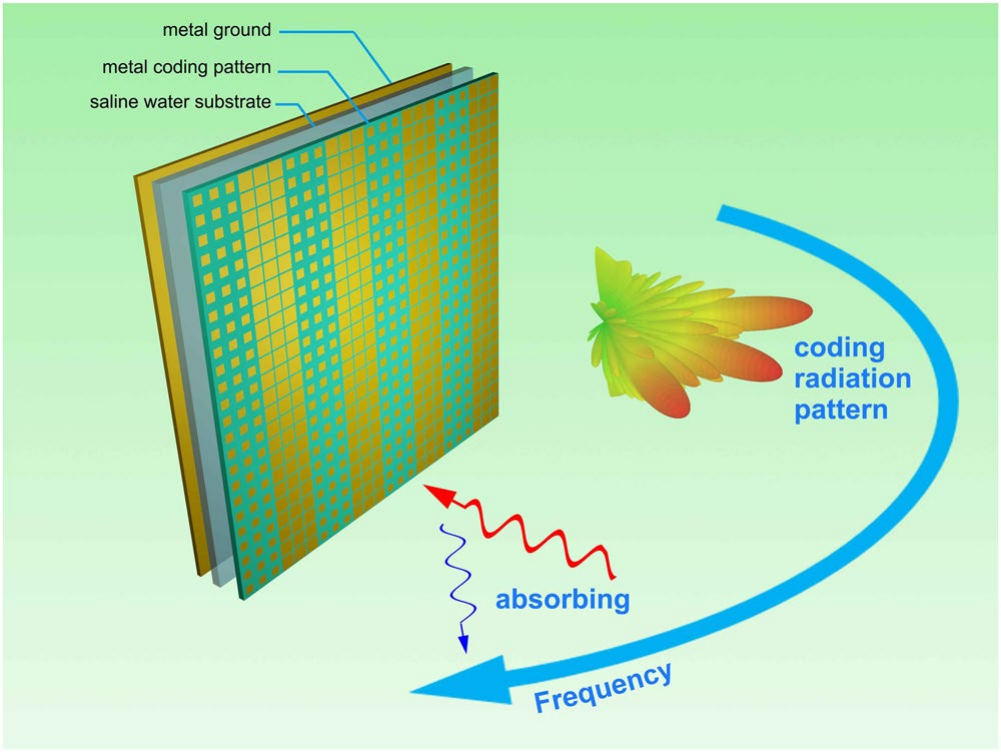
Schematic of bifunctional tunable coding metasurface based on water substrate. Specific coding radiation patterns and absorption are realized in different frequency. The absorbing ratio can be manipulated by the salinity in water substrate. The designed coding metasurface is composed of three layers, F4B with metal coding pattern, saline water substrate and metal ground.

To verify our idea, a new coding metasurface with two functions is designed as Fig. 1 showed. The whole metasurface is composed of three layers, which are F4B substrate with specific metal coding pattern, solution substrate and background metal board respectively. The two functions, specific coding radiation pattern and absorbing character, can be selected in frequency domain. Besides, since the salinity can effectively influence the imaginary part of water permittivity, the absorbing performance can also be tuned by changing the salinity of the solution substrate.

**Figure 2:**
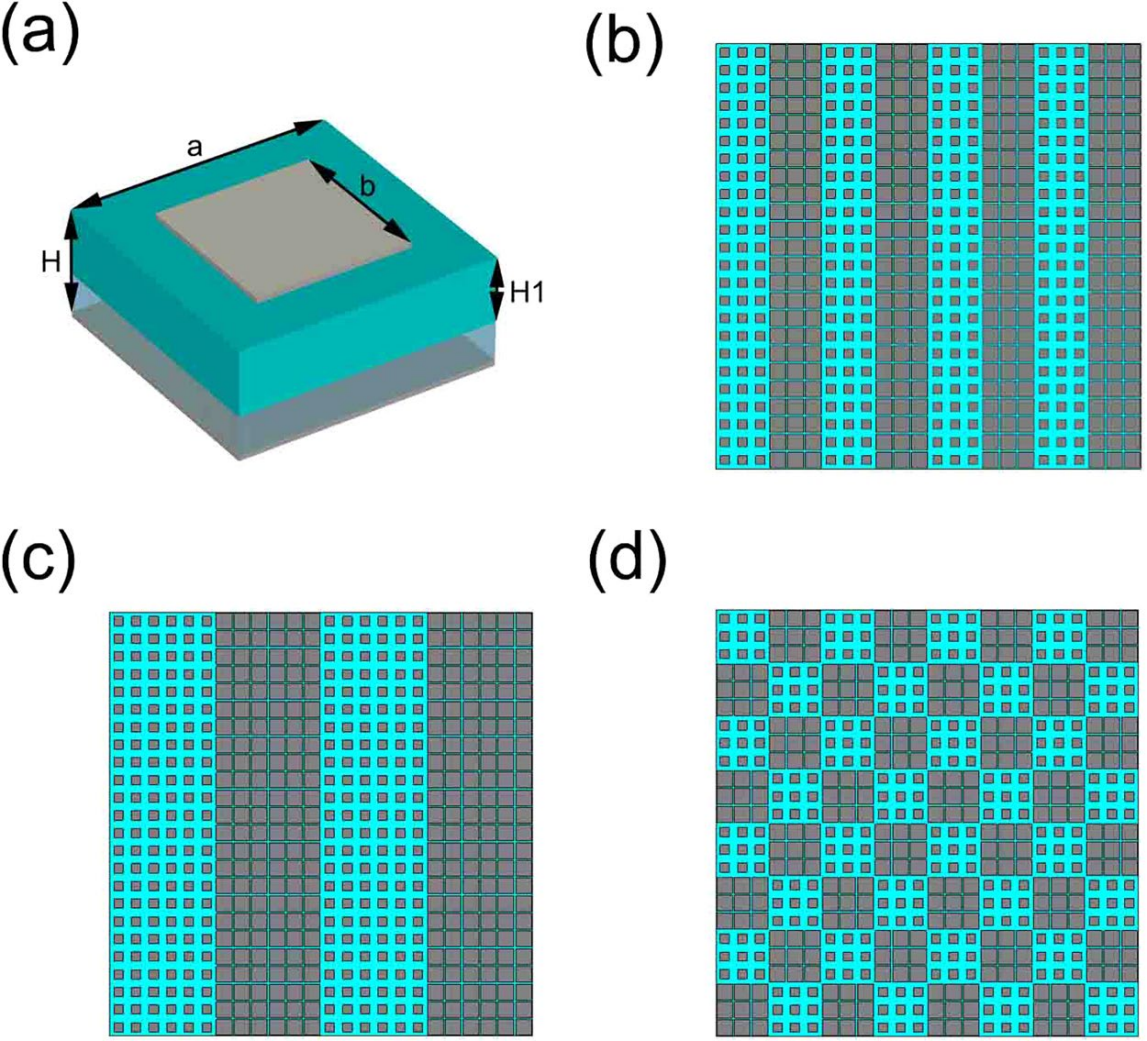
The structure of unit cell and three designed coding patterns. (**a**) The structure of elements, with relevant dimension marks. (**b**) The designed coding pattern array of sequence “01010101”. (**c**) The designed coding pattern array of sequence “00110011”. (**d**) The designed coding pattern array of chess board.

To realize these specialties in single module, a simple unit cell is employed as Fig. 2(a) showed. The structures from the top to the bottom are, F4B (with a dielectric constant of 2.65 and loss tangent of 0.001) substrate with square metallic patch on it, solution substrate and metallic ground. The related structure sizes are marked in Fig. 2(a), in which *H* means the total height of the element and *H1* means the height of F4B substrate. *H* and *H1* are set as 2 mm and 1.2 mm respectively, and *a* is 10 mm. Two dimensions of *b* selected are 5.4 mm and 8.2 mm, considered as digital “0” and “1” respectively, whose phase responses are nearly 0° and 180° at 7 GHz. Note that in our coding scheme, each supercell contained 3 * 3 elements is considered as a code. And based on these supercells, three coding patterns of metasurfaces are designed to be “01010101”, “00110011” and chess board array respectively, as Fig. 2(b),(c) and (d) showed. The quadrate array is composed of 8 * 8 supercells (24 * 24 elements), with the size of 240 * 240 mm.

**Figure 3:**
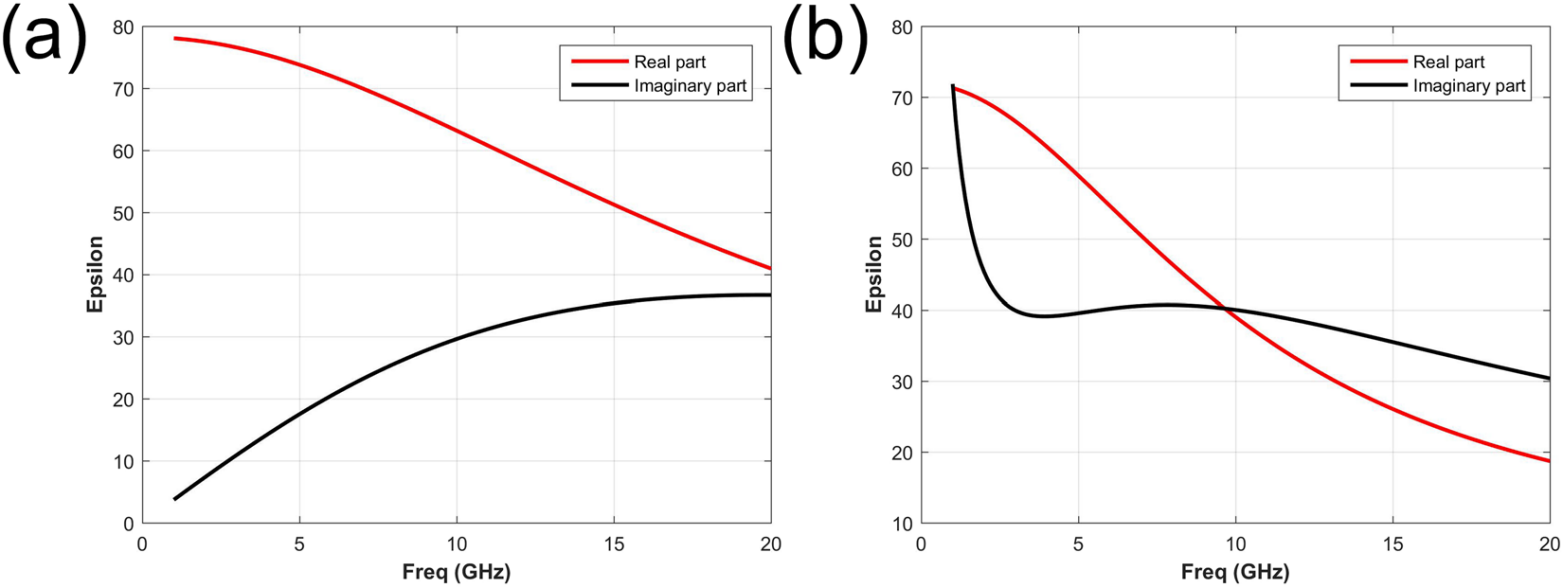
The complex permittivities of pure water and NaCl solution. (**a**) The complex permittivity of pure water. (**b**) The complex permittivity of 30‰ NaCl solution. The real part and imaginary part are noted with red and black color respectively.

To verify the tunability of the salinity, pure water and NaCl solution with salinity of 30‰ are chosen, and the temperature is set at 25 °C.The permittivity of water and NaCl solution is calculated by the Debye formula^17^. Figure 3(a) and (b) shows the complex permittivity of pure water and NaCl solution, the real part and imaginary part are noted with red and black color respectively. The dramatic difference in frequency dispersion can be observed between water and NaCl solution, which provides a new degree of freedom to control the reflective properties of metasurfaces.

**Figure 4:**
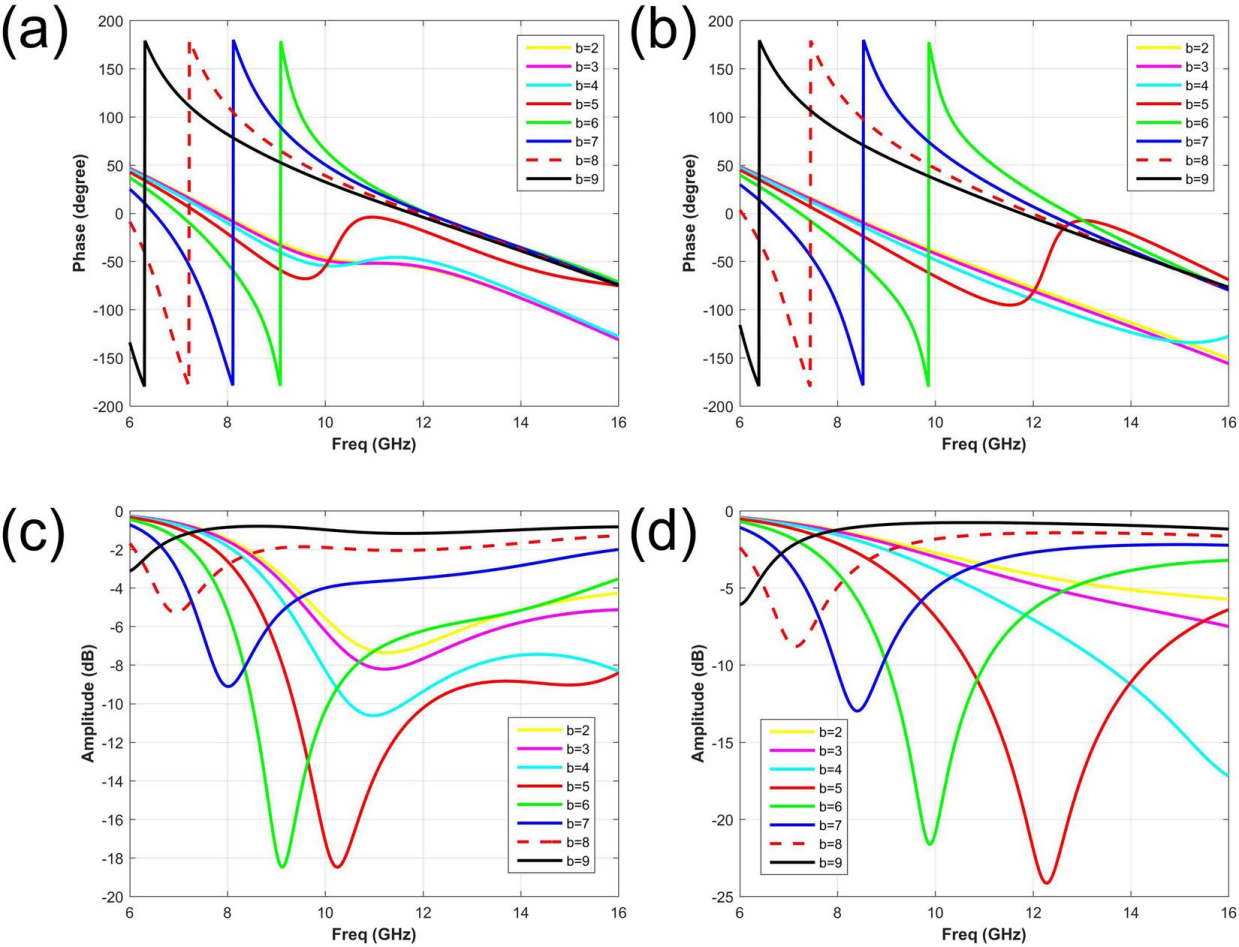
The phase and amplitude responses of elements with different dimension *b*, based on the pure water substrate and NaCl solution substrate respectively. (**a**) and (**b**) The phase responses of elements based on pure water and NaCl solution substrate respectively. (**c**) and (**d**) The amplitude responses of elements based on pure water and NaCl solution substrate respectively. The results of *b* = 2, 3, 4, 5, 6, 7, 8, 9 mm are noted with different colors and linetypes.

**Figure 5:**
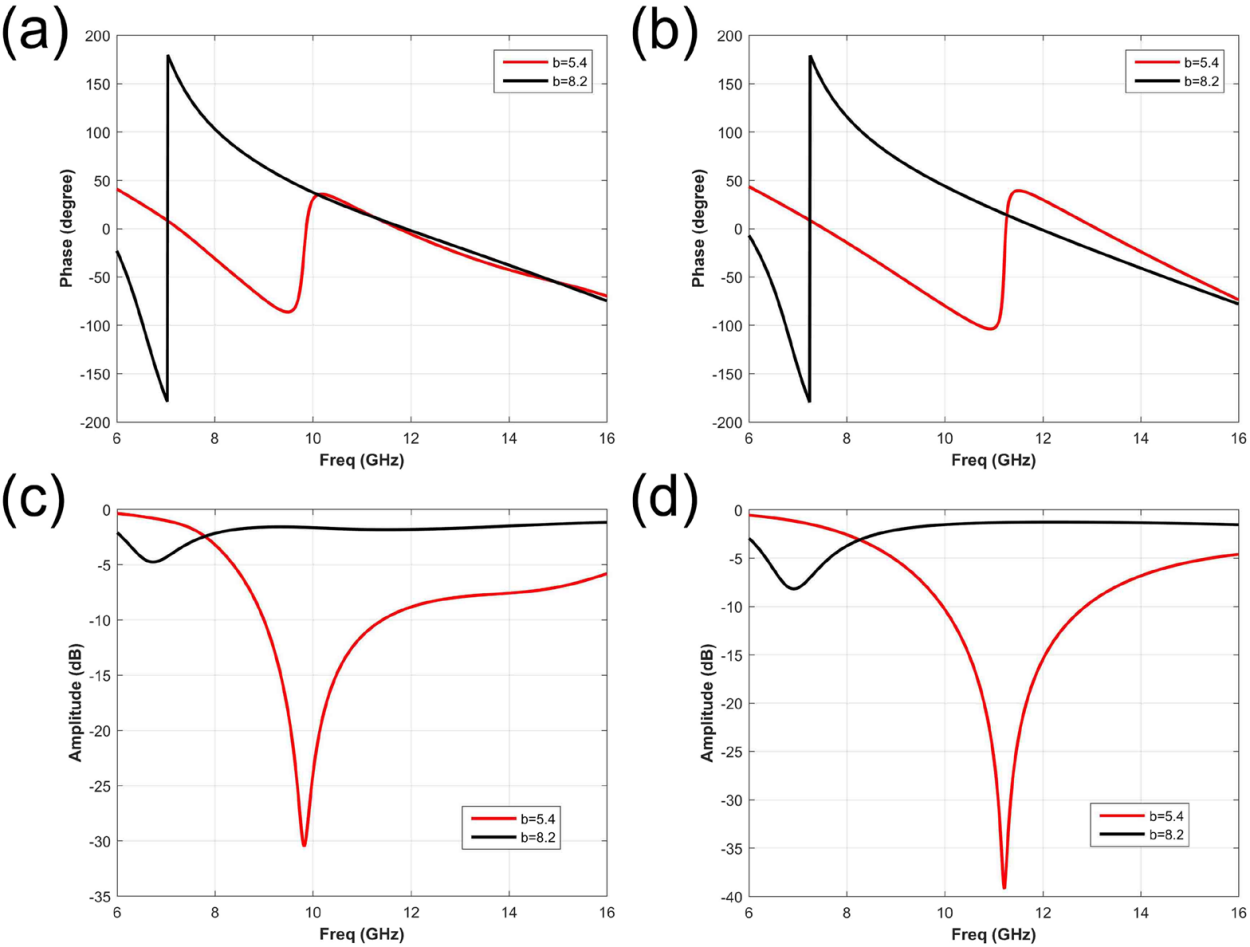
The phase and amplitude responses of two selected elements, with *b* = 5.4 and 8.2 mm. (**a**) and (**b**) The phase responses of elements based on pure water and NaCl solution substrate respectively. (**c**) and (**d**) The amplitude responses of elements based on pure water and NaCl solution substrate respectively. The data for a = 5.4 and 8.2 mm are noted with red and black color respectively.

The simulations are performed in the commercial software, Computer Simulation Technology (CST) Microwave Studio. The phase and amplitude response results are illustrated in Fig. 4. The phase and amplitude responses of pure water substrate, with different sizes of *b* (from 2 mm to 9 mm) are given in Fig. 4(a) and (c) respectively. The relative results of saline water substrate are showed in Fig. 4(b) and (d). With different salinities (0‰ and 30‰) in water substrate, both the phase responses and the resonance points with different dimensions of b experience different frequency shifts. By selecting appropriate size of *b*, absorbing property in high frequency can be altered by salinity, and keep the coding radiation pattern in low frequency unchanged simultaneously. In other words, the reflective amplitude can be manipulated easily with the salinity. Based on the above simulation results, two elements of *b* = 5.4 mm and 8.2 mm are selected as digital “0” and “1”, whose phase reflections are approximately 0° and 180° at 7 GHz. The relevant simulation consequences are listed in Fig. 5(a) and (b), which represents the element with pure water and saline water substrate respectively. For tunable absorbing function, simulated consequences are given in Fig. 5(c) and (d), from which the reflective magnitude of *b* = 5.4 mm is varied at 10 GHz with different salinities. On the contrary, the magnitude response of the element with *b* = 8.2 mm remains the same.

**Figure 6:**
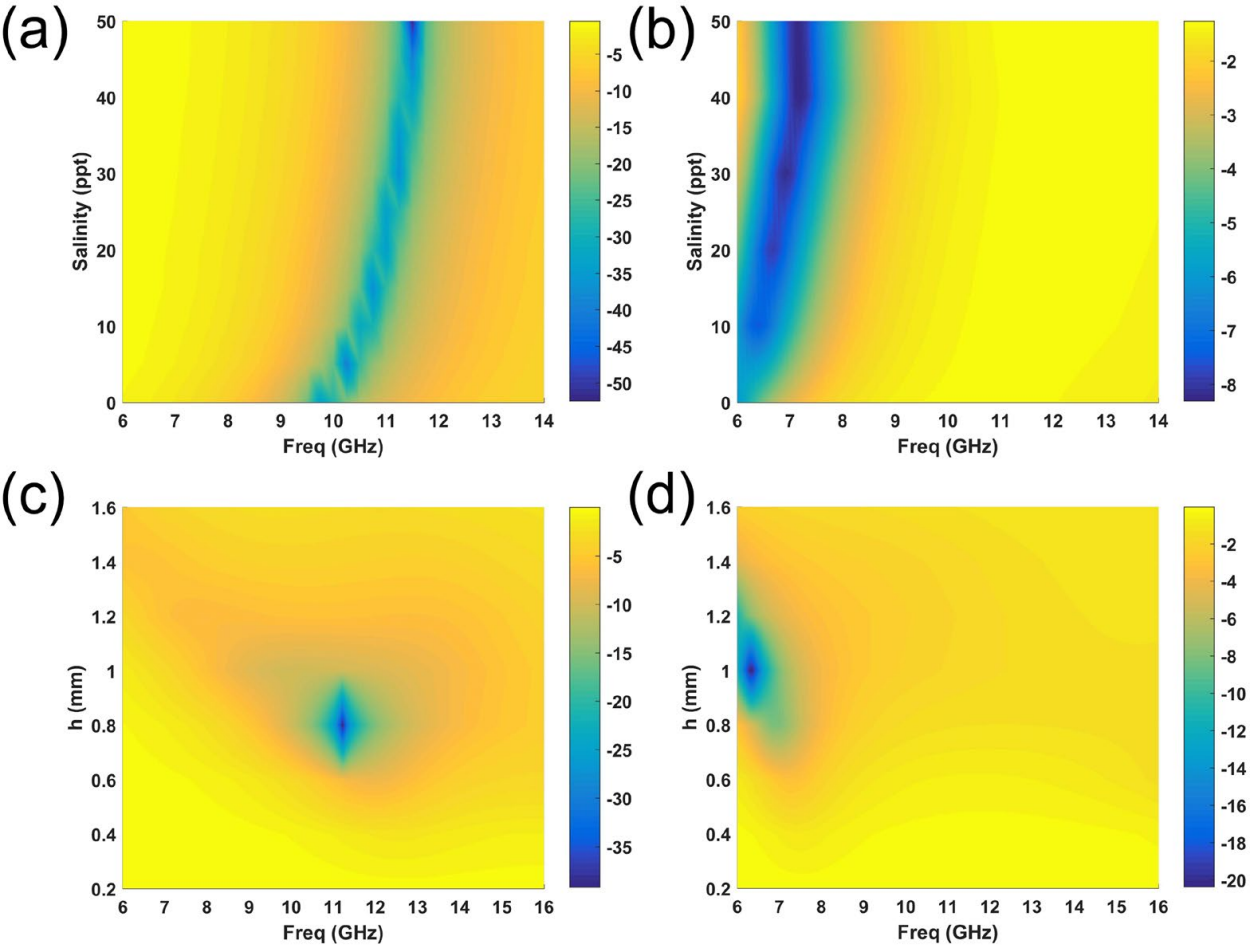
(**a**) The salinity dependence of the simulated S11 for the element with *H1* = 1.2 mm and *b* = 5.4 mm. (**b**) The salinity dependence of the simulated S11 for the element with *H1* = 1.2 mm and *b* = 5.4 mm. (**c**) The solution-substrate thickness dependence of the simulated S11for the element with 30‰ salinity and *b* = 5.4 mm. (**d**) The solution-substrate thickness dependence of the simulated S11 for the element with 30‰ salinity and *b* = 8.2 mm.

To further analyze the relation among the frequency resonance, salinity and the thickness of solution substrate, numerous simulations are performed as Fig. 6 showed. The simulated S11 of two elements (b = 5.4 mm and 8.2 mm) are given in Fig. 6(a) and (b), to reveal the salinity dependence of S11 for the elements with *H1* = 1.2 mm. From the results, the obvious resonance shifts can be observed from 9.8 GHz to 11.5 GHz. Figure 6(c) and (d) show the solution-substrate thickness *h* dependence of S11 for the two elements (b = 5.4 mm and 8.2 mm) with 30‰ salinity. From the data, there is no resonance shift can be observed. The slight discontinuity in the Fig. 6 is mainly due to the discretization of simulated data. According to the results from Figs 4 and 6, the resonance frequency of this element is mainly determined by the dimension *b* and salinity.

**Figure 7:**
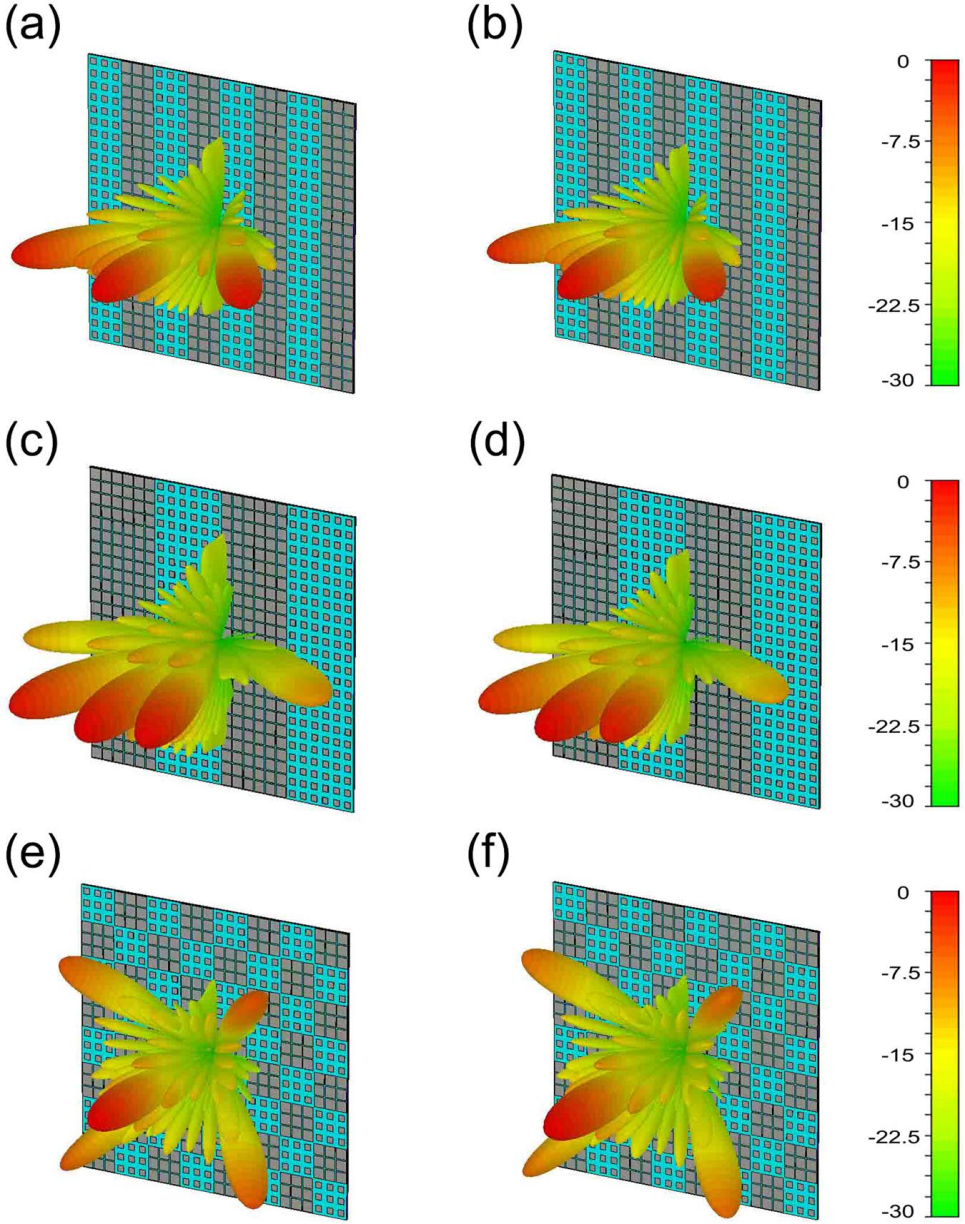
The far-field radiation pattern of three coding sequences based on pure water substrate and NaCl solution substrate respectively. (**a**) and (**b**) The radiation patterns of coding sequence “01010101” based on pure water substrate and NaCl solution substrate respectively. (**c**) and (**d**) The radiation patterns of coding sequence “00110011” based on pure water substrate and NaCl solution substrate respectively. (**e**) and (**f**) The radiations pattern of chess board arrays based on pure water substrate and NaCl solution substrate respectively.

The EM full-wave simulations of the three coding pattern arrays are also performed in CST Microwave Studio. Figure 7 provides the simulated far-field results at 7 GHz. (a), (c) and (e) are the arrays with pure water substrate, while (b), (d) and (f) are the arrays with NaCl solution substrate. The results of coding sequence “01010101” are listed in Fig. 7(a) and (b), three beams with wide deflected angle can be observed clearly. The array of sequence “00110011” generates three beams with narrow angle, as Fig. 7(c) and (d) showed. And the radiation pattern of chess board array in Fig. 7(e) and (f) contains five beams. Apparently, in our three coding sequences, the coding radiation patterns of disparate salinities are quite similar, verifying our designs.

**Figure 8:**
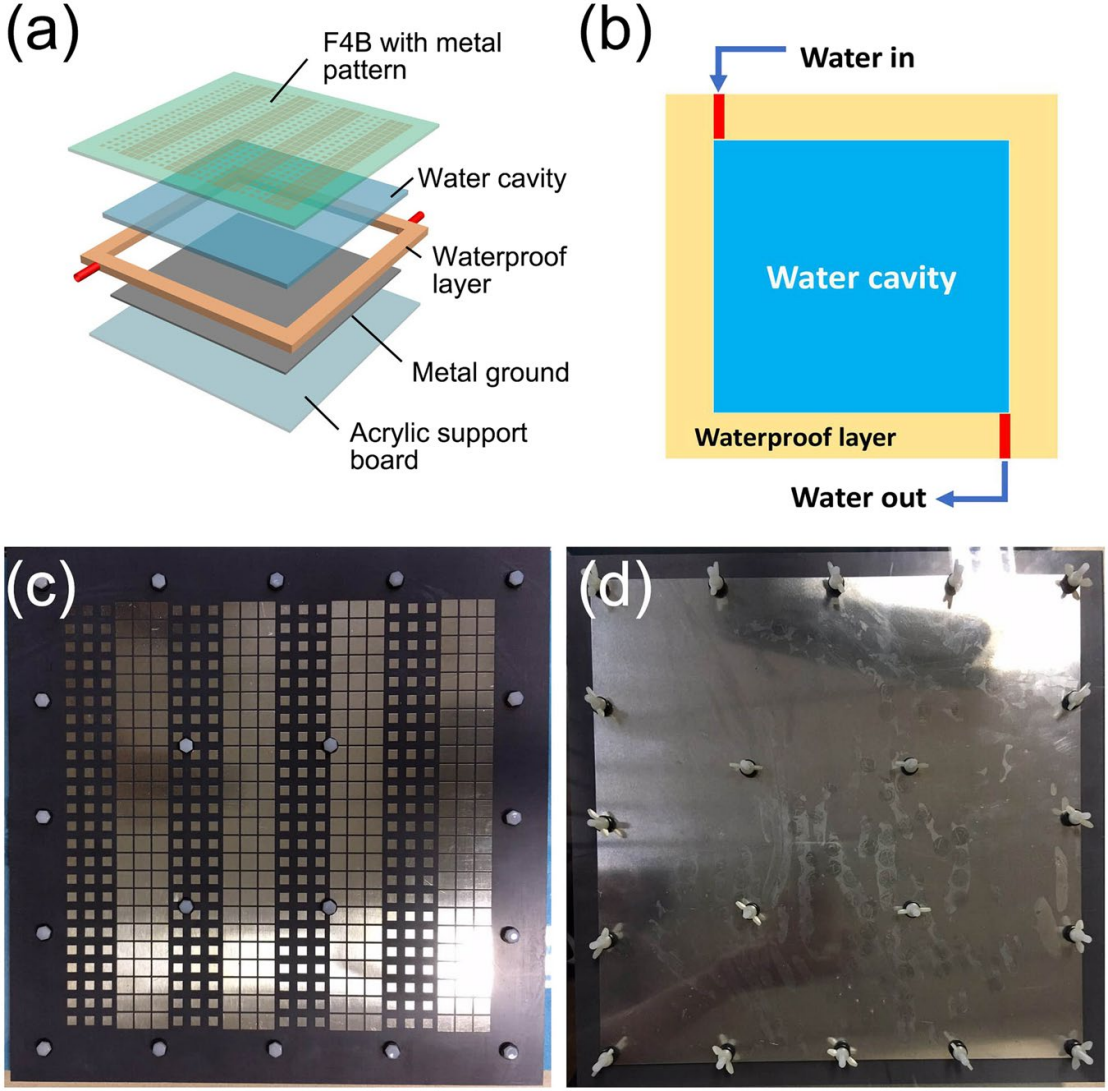
The structure schematic and the fabricated sample of coding sequence “01010101”. (**a**) The detailed structure of the fabricated sample. (**b**) The illustration of water exchange in the water cavity. (**c**) The top view of the fabricated sample. (**d**) The bottom view of the fabricated sample.

In the experimental demonstration, one of three coding sequences, “01010101” is manufactured. Printed Circuit Board (PCB) technology is applied to fabricate the specific metal pattern on F4B substrate, with the dimension of 300 mm * 300 mm. The water cavity between F4B substrate and metal ground, with thickness of 0.8 mm, is supported by the screws all around as Fig. 8 showed. Figure 8(a) illustrates the detailed structure of the fabricated sample. The water cavity between metal ground and top F4B layer, is supported by gaskets. The whole cavity is surrounded closely by a waterproof layer, which is composed of polyvinyl chloride (PVC). The sandwich is adhered with the epoxy resin, and is further tightened with plastic bolts on the edge. So the saline water can be safely contained in the designed metasurface. Two small pipes are reserved for water exchange. Figure 8(b) reveals the schematic of water exchange in water cavity. The top and bottom view of the fabricated sample is showed in Fig. 8(c) and (d).

**Figure 9:**
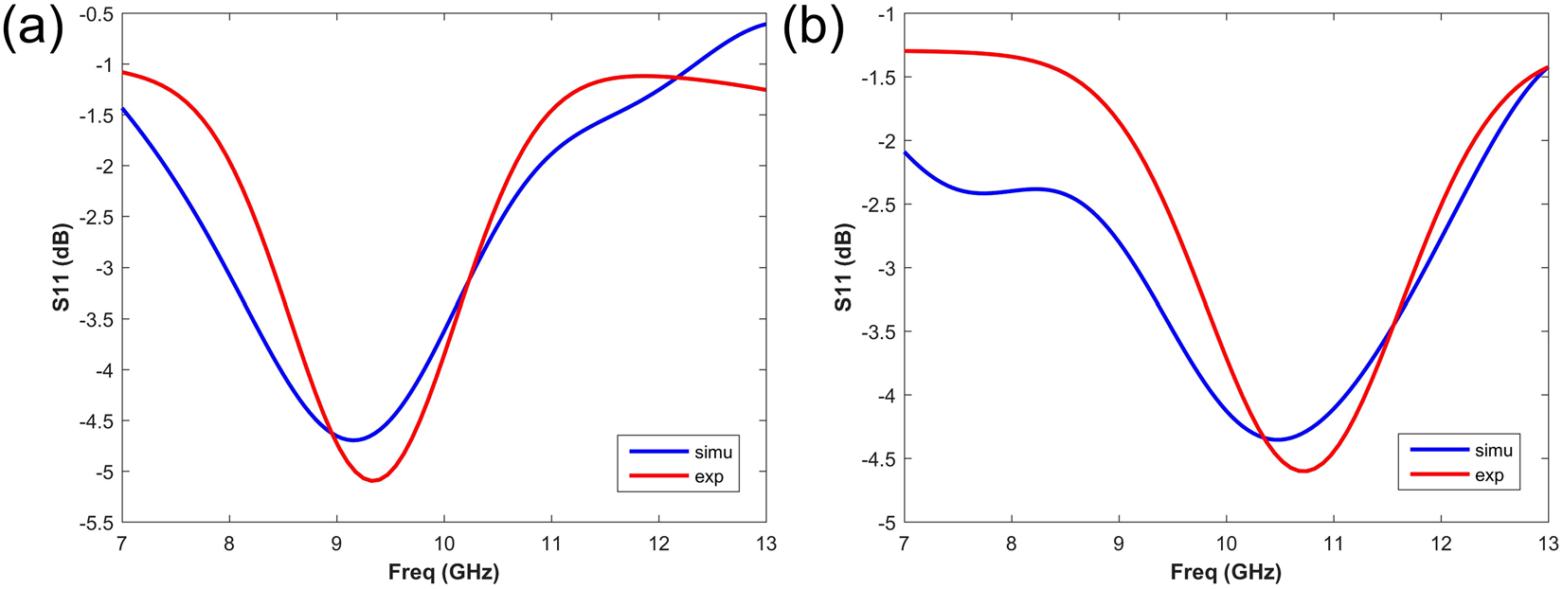
The simulated and measured absorption performance (S11) of the array with coding sequence “01010101”. (**a**) The comparison between simulation and measurement of coding pattern “01010101” based on the pure water substrate. (**b**) The comparison between simulation and measurement of coding pattern “01010101” based on the NaCl solution substrate. The simulated and measured results are marked with blue and red color respectively.

To prove the tunable absorbing performance based on salinity, the comparison between simulations and measurements is given in Fig. 9. The results of pure water substrate and NaCl solution substrate are given in Fig. 9(a) and (b) respectively. The simulated and the measured data are marked with blue and red color respectively. The distinctive variation absorbing capability can be observed, as the salinity of the water substrate varies. With the pure water substrate, the designed array realizes about −5 dB absorption at 9 GHz. As the salinity varies from 0‰ to 30‰, the resonance point moves from 9 GHz to 10.8 GHz. The measured efficiency of fabricated metasurface with water-substrate is 72% at 7 GHz and 30% at 9 GHz. The efficiency of metasurface with water-substrate is 69% at 7 GHz and 32% at 10.8 GHz. The simulated results show great consistency with measured results, slight deviation is mainly to the imprecise fabrications.

**Figure 10:**
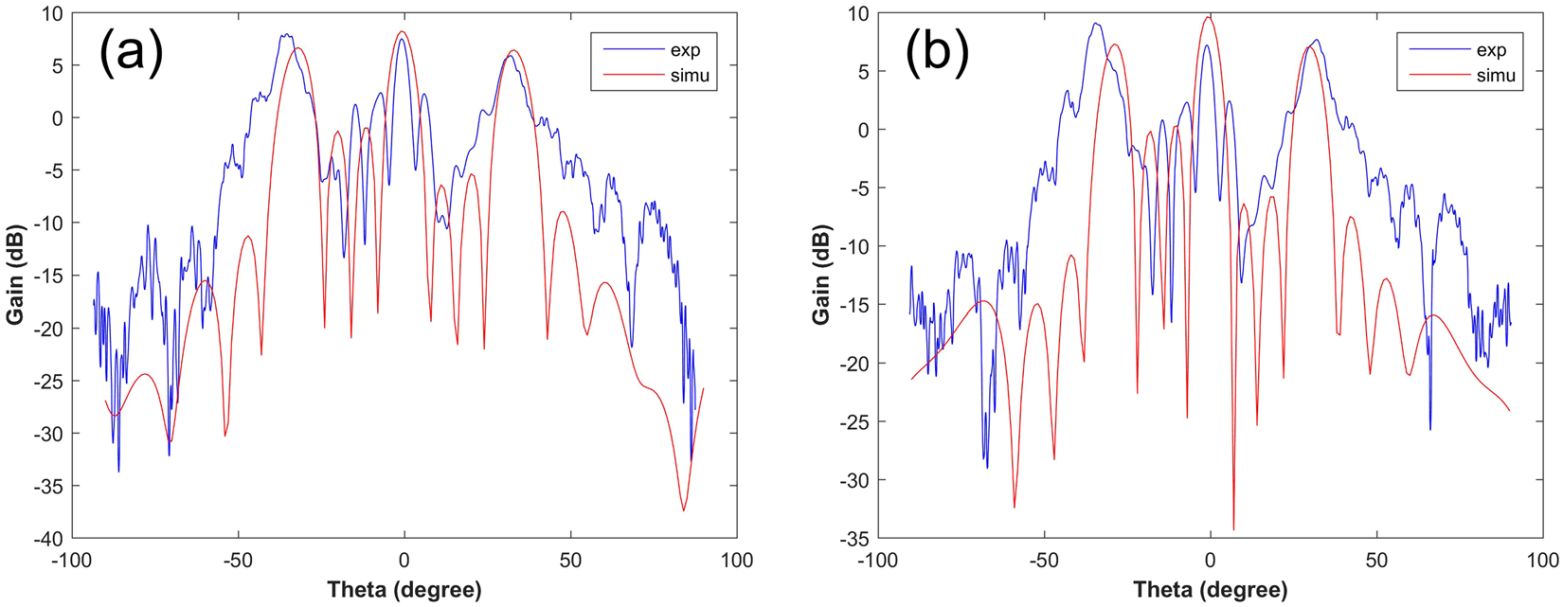
The simulated and measured far-field results of the array with coding sequence “01010101”. (**a**) The comparison between simulation and measurement of coding pattern “01010101” based on the pure water substrate. (**b**) The comparison between simulation and measurement of coding pattern “01010101” based on the NaCl solution substrate. The simulated and measured results are marked with blue and red color respectively.

The far-field results are given in Fig. 10(a) and (b) are the results based on the pure- and saline-water substrate. The three emitting beams are detected clearly, which shows good agreement with the simulations. The minor measurement error between the simulated and the measured is owing to the manual operation in experiments and manufacture error. The far-field patterns of pure water substrate and solution substrate also show good similarity, further demonstrating the accuracy of our designs.

## Discussion

In a word, we propose a new tunable coding metasurface integrated two functionalities, which can be selected in frequency domain. Using water substrate, specific coding radiation patterns and tunable absorption is achieved in low and high frequency respectively. As a new degree of freedom for manipulation, salinity in water substrate is employed to tune the resonance status. Pure water and NaCl solution are selected as two different salinity substrates, to demonstrate our schemes. Three coding sequence arrays are proposed, and one of them is manufactured for experimental verification. The measurement results reveal great uniformity with simulations, for both two functionalities. We expect this work will arise more enthusiasm on the water-based metasurface and be extended to further practical fields like biological and medical diagnosis.

## Methods

Numerical simulations are performed by commercial software, the CST Microwave Studio. To realize two functions simultaneously in a single metasurface, we design an element with metal patch and water substrate. The element simulations of parameter sweep are performed, to obtain the most appropriate dimensions, which can realize 180° phase difference at 7 GHz and tunable absorption at 9 GHz simultaneously. The two dimension of *b* are chosen as 5.4 mm and 8.2 mm, to represent digital “0” and “1” respectively.

The experimental sample is fabricated on a 1.5 mm-thick F4B substrate with permittivity 2.65 and loss tangent 0.001. The water cavity is between F4B and metal ground, whose thickness is set as 0.8 mm. PCB technology is applied to fabricate the specific metal pattern on F4B substrate, with the dimension of 300 mm * 300 mm. The coding array is composed of 24 * 24 unit cells, occupied a 240 mm * 240 mm area. In the absorption measurements, a broadband rectangular horn is applied to test S11. The Agilent vector network analyzer is employed to measure S-parameter. The reflection from a copper plate with same size (300 mm * 300 mm) is measured firstly for the calibration. Then the coding metasurfaces with pure water and NaCl solution substrate are tested, to obtain the S11 data. In the far-field experiments, two broadband rectangular horns are used as the transmitting terminal and receiving terminal respectively.
